# Changes in glutamate levels in anterior cingulate cortex following 16 weeks of antipsychotic treatment in antipsychotic-naïve first-episode psychosis patients

**DOI:** 10.1017/S0033291724003386

**Published:** 2025-02-10

**Authors:** Jose Maximo, Eric Nelson, Nina Kraguljac, Rita Patton, Adil Bashir, Adrienne Lahti

**Affiliations:** 1 Department of Psychiatry and Behavioral Neurobiology, Heersink School of Medicine, University of Alabama at Birmingham, Birmingham, AL, USA; 2 Department of Psychiatry and Behavioral Health, College of Medicine, Ohio State University, Columbus, OH, USA; 3 Department of Electrical and Computer Engineering, Samuel Ginn College of Engineering, Auburn University, Auburn, AL, USA

**Keywords:** antipsychotic medication-naïve, first-episode psychosis, glutamate, longitudinal, magnetic resonance spectroscopy, treatment response

## Abstract

**Background:**

Previous findings in psychosis have revealed mixed findings on glutamate (Glu) levels in the dorsal anterior cingulate cortex (dACC). Factors such as illness chronicity, methodology, and medication status have impeded a more nuanced evaluation of Glu in psychosis. The goal of this longitudinal neuroimaging study was to investigate the role of antipsychotics on Glu in the dACC in antipsychotic-naïve first-episode psychosis (FEP) patients.

**Methods:**

We enrolled 117 healthy controls (HCs) and 113 antipsychotic-naïve FEP patients for this study. 3T proton magnetic resonance spectroscopy (1H-MRS; PRESS; TE = 80 ms) data from a voxel prescribed in the dACC were collected from all participants at baseline, 6, and 16 weeks following antipsychotic treatment. Glutamate levels were quantified using the QUEST algorithm and analyzed longitudinally using linear mixed-effects models.

**Results:**

We found that baseline dACC glutamate levels in FEP were not significantly different than those of HCs. Examining Glu levels in FEP revealed a decrease in Glu levels after 16 weeks of antipsychotic treatment; this effect was weaker in HC. Finally, baseline Glu levels were associated with decreases in positive symptomology.

**Conclusions:**

We report a progressive decrease of Glu levels over a period of 16 weeks after initiation of treatment and a baseline Glu level association with a reduction in positive symptomology, suggestive of a potential mechanism of antipsychotic drug (APD) action. Overall, these findings suggest that APDs can influence Glu within a period of 16 weeks, which has been deemed as an optimal window for symptom alleviation using APDs.

## Introduction

Schizophrenia (SZ) is a heterogeneous disorder that likely involves multiple underlying pathological mechanisms (Joyce & Roiser, 2007), which has plagued attempts to identify rational therapeutic targets (Liang & Greenwood, 2015). All current antipsychotic drugs (APD) are dopamine receptor antagonists, but the clinical response is variable, with one-third of patients being partial responders and another third nonresponders (Harrow, Sands, Silverstein, & Goldberg, 1997). In addition, there is also a group of patients who experience a delayed treatment response, with response still accruing between 8 and 16 weeks after treatment initiation (Gallego et al., 2011). Arguably, those who respond well to APD have primarily dopaminergic abnormalities (Abi-Dargham et al., 1998; Abi-Dargham et al., 2000), but it is imperative to also characterize the specific underlying pathologies in those with poor or delayed response in order to unravel the heterogeneity of psychosis and effectively develop new treatments.

Disruption in glutamatergic function has been considered an important part of the pathophysiology of psychosis since the demonstration that acute administration of the N-methyl-d-aspartate glutamate receptor (NMDAR) antagonist ketamine can induce psychosis-like symptoms in healthy individuals (Lahti, Weiler, Tamara Michaelidis, Parwani, & Tamminga, 2001; Malhotra et al., 1996) and exacerbate psychosis in patients with SZ (Lahti, Holcomb, Medoff, & Tamminga, 1995; Malhotra et al., 1997). Furthermore, postmortem findings in psychotic patients such as abnormal glutamate (Glu) in cerebrospinal fluid (Kim, Kornhuber, Schmid-Burgk, & Holzmuller, 1980) and brain tissue (Hu, MacDonald, Elswick, & Sweet, 2015) have provided additional evidence of abnormal glutamatergic functioning in psychosis. In vivo proton magnetic resonance spectroscopy (1H-MRS) has provided an excellent avenue to characterize Glu in psychosis (Kruse & Bustillo, 2022).

Converging evidence from postmortem (Roberts et al., 2022), multimodal brain imaging (Overbeek, Gawne, Reid, Kraguljac, & Lahti, 2021) and neuropsychological assessments (Minzenberg, Laird, Thelen, Carter, & Glahn, 2009) indicate that anterior cingulate cortex (ACC) dysfunction is central to the pathophysiology of SZ. Since this region has been shown to be modulated by APDs in a clinically relevant fashion (Lahti, Weiler, Holcomb, Tamminga, & Cropsey, 2009), it has been implicated in a neuronal circuit mediating antipsychotic action. Furthermore, neuroimaging studies have shown that the effects of ketamine are mediated in anterior cingulate regions (Deakin et al., 2008; Lahti, Holcomb, Medoff, & Tamminga, 1995).

Our group previously reported no significant group differences in glutamate + glutamine (Glx) in the dorsal ACC (dACC) on a large sample of antipsychotic-naïve first-episode psychotic (FEP) patients compared to healthy controls (HCs) prior to APD treatment (Maximo, Briend, Armstrong, Kraguljac, & Lahti, 2021). Two recent meta-analysis reporting on a very large number of patients found lower medial frontal cortex (MFC) Glu in patients compared to HCs, but stratified analyses indicated that this was not the case in antipsychotic-naïve/unmedicated patients (Merritt et al., 2023; Smucny, Carter, & Maddock, 2021).

Several longitudinal studies in SZ (Bojesen et al., 2020; Bustillo et al., 2010; Egerton et al., 2018; Fan et al., 2024; Jeon et al., 2021; Kraguljac et al., 2019; Wang et al., 2023; Zahid et al., 2022) examined the effect of APD in dACC Glu after a period of 2, 4, 6, 12 weeks, and 4 years following treatment. These studies included mixed samples of minimally treated and medication-naïve patients and relatively small to medium sample sizes (n’s = 14–61). Six of these studies did not show a significant change in ACC Glu levels over time (Bojesen et al., 2020; Bustillo et al., 2010; Fan et al., 2024; Jeon et al., 2021; Kraguljac et al., 2019; Zahid et al., 2022) and only two reported reductions in Glu in patients (Egerton et al., 2018; Wang et al., 2023).

Importantly, some studies identified associations between baseline level of ACC Glu and treatment response or remission status, but their results diverge. Egerton et al. (2018) reported that higher ACC Glu/Creatine (Glu/Cr) at baseline was associated with a lower likelihood of remission after 4 weeks. On the other hand, Li et al. (2020) found the opposite: nonremission patients (as assessed after 8 weeks) had lower baseline ACC Glu. In chronically ill treatment-resistant patients compared with treatment-responsive patients, Glu/Cr levels were higher in treatment-resistant patients (Mouchlianitis et al., 2016). Lastly, Dempster et al. (2020) and Yang et al. (2022) found no differences between remission status in patients. Given these inconsistencies, there is a need for well-powered studies to elucidate the baseline and trajectory of Glu levels in medication-naïve FEP.

The goal of this prospective longitudinal 1H-MRS study was to assess Glu in the dorsal ACC (dACC) in a large sample of antipsychotic-naïve FEP throughout an antipsychotic medication trial by collecting Glu prior to treatment and after 6 and 16 weeks of treatment. This time span allowed us to cover the full extent of time to respond. We also collected 1H-MRS data for matched HC for the same period. We then assessed treatment response as measured in decreases of positive symptomology in FEP patients with baseline and Glu changes, as well as by contrasting treatment remitters’ versus nonremitters using the Andreasen criteria (Andreasen et al., 2005). Based on our own findings (Maximo, Briend, Armstrong, Kraguljac, & Lahti, 2021), we did not expect to find differences in baseline Glu between FEP and HC. Based on prior literature (Egerton et al., 2018; Merritt et al., 2023; Merritt et al., 2021), we hypothesized that there will be decreases in Glu after 6 weeks and after 16 weeks of antipsychotic treatment (and no changes in HCs) and that baseline and changes in Glu levels will be negatively associated with treatment response in medication-naïve FEP patients.

## Materials and methods

### Participants

A total of 230 (HC = 117; FEP = 113) participants with available neuroimaging data were included in this study. Antipsychotic naive FEP patients were recruited from the emergency room, inpatient units, and outpatient psychiatry clinics at the University of Alabama at Birmingham (UAB). Studies were approved by the University of Alabama at Birmingham Institutional Review Board, and written informed consent was obtained before enrollment [patients had to have the capacity to provide consent (Carpenter et al., 2000)]. Diagnoses were established by consensus of two board-certified psychiatrists (ACL and NVK) taking into consideration information from the Diagnostic Interview for Genetic Studies (DIGS) or Mini-International Neuropsychiatric Interview (MINI) and medical records as available. In addition, because of the longitudinal design of the study (ClinicalTrials.gov Identifier: NCT02034253, NCT03442101), clinical observations over several months of follow-up were used to establish a final diagnosis. The Brief Psychiatric Rating Scale was used to assess symptom severity (Overall & Gorham, 1962). Cognitive function was characterized using the Repeatable Battery for the Assessment of Neuropsychological Status (RBANS) (Randolph, Tierney, Mohr, & Chase, 1998). Exclusion criteria for patients were major neurological or medical conditions, history of significant head trauma, substance-use disorders (excluding nicotine and cannabis) within 1 month of imaging, patients with very limited exposure of no more than 5 days of lifetime antipsychotic exposure, pregnancy or breastfeeding, and MRI contraindications. Use of concomitant medications, including antidepressants, was permitted as clinically indicated and was prescribed after the MRI session. We did not exclude patients based on a prespecified maximal duration of symptoms before study entry. We also recruited HCs who were matched on age, sex, and parental socioeconomic status (SES). In addition to the above outlined criteria, HCs with a personal history or a family history of a psychiatric illness in a first-degree relative were also excluded.

FEP patients enrolled in this longitudinal study entered a 16-week trial of oral risperidone using a flexible dosing regimen. We chose risperidone because it is commonly prescribed, now available as a generic medication and thus one of the more affordable second-generation antipsychotic medications in the United States and is considered a first-line treatment in FEP (Robinson et al., 2015). Risperidone was started at 0.5–1 mg and titrated in 1–2 mg increments; dosing was based on therapeutic and side effects. In case of excessive side effect burden, as determined by a study physician, patients were switched to aripiprazole started at 2–5 mg and titrated in 2.5–10 mg increments (four patients were switched to aripiprazole). Use of concomitant medications, including antidepressants, was permitted as clinically indicated and was prescribed after the initial MRI session. Twenty-two medication-naïve FEP were prescribed the following during the trial: trazodone (3), sertraline (10), valproic acid (1), bupropion (1), escitalopram (1), lorazepam (1), suboxone (1), fluoxetine (1), citalopram (1), propranolol (1), and amitriptyline (1). Seventeen other patients were under more than one medication. Compliance was monitored with pill counts at each visit.

### Clinical assessment

First, the percent BPRS Positive-4 score reduction (hallucinatory behavior, unusual thought content, suspiciousness, and conceptual disorganization) was calculated after subtraction of minimum possible scores (Leucht, Davis, Engel, Kissling, & Kane, 2009) and a greater percentage indicates a greater reduction in positive symptoms. Treatment response data were available for 84 patients.

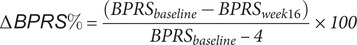



Then, treatment outcomes were also evaluated with the Andreasen criteria, in which remission is defined as a score of mild or less (< 3) on the following 6 BPRS scores: grandiosity, suspiciousness, unusual thought content, hallucinatory behavior, conceptualized disorganization, mannerism, and blunted effect (Andreasen et al., 2005).

### Data acquisition

All imaging was performed on a 3T whole-body Siemens MAGNETOM Prisma MRI scanner equipped with a 20-channel head coil. A high-resolution T1-weighted structural scan was acquired for anatomical reference (MPRAGE: TR = 2400 ms; TE = 2.22 ms; inversion time = 1000 ms; flip angle = 8°; GRAPPA factor = 2; voxel size = 0.8 mm3). 1H-MRS data were collected from a voxel in the dorsal ACC (27 × 20 × 10 mm^3^, Figure 2A). Following automatic and manual shimming to optimize field homogeneity across the voxel, chemical shift selective (CHESS) pulses were used to suppress the water signal. Then, spectra were obtained using a point resolved Spectroscopy sequence (PRESS; TR/TE = 2000/80 ms, flip angle = 90°, vector size 1024, 96 averages) (Mullins, Chen, Xu, Caprihan, & Gasparovic, 2008; Schubert, Gallinat, Seifert, & Rinneberg, 2004). Moreover, eight averages of unsuppressed water scans with the same acquisition parameters were acquired as an internal reference.

### MRS preprocessing

All spectra were preprocessed in jMRUI version 6.0 using the QUEST algorithm (Ratiney, Coenradie, Cavassila, van Ormondt, & Graveron-Demilly, 2004; Ratiney et al., 2005). The basis set was simulated using the timing parameters of the PRESS sequence. The simulation consisted of peaks for N-acetyl aspartate (NAA), choline (Cho), creatine (Cr), and glutamate (Glu). Glutamine was also included in our basis set as a metabolite of no interest separate from glutamate to increase the quality of the glutamate signal. The position (frequency) and linewidths of individual metabolites were independently adjusted to fit the data and the Subtract approach was used for background handling. After removing the residual water peak using the Hankel–Lanczos singular values decomposition filter, the metabolite amplitudes for NAA, Cho, Cr, and Glu were estimated and then calculated relative to the unsuppressed voxel water and expressed in institutional units (Scheidegger et al., 2013). Voxel tissues were segmented using the Gannet toolbox (version 3.1) (Edden, Puts, Harris, Barker, & Evans, 2014). Metabolite levels were then corrected for partial volume effects according to Gasparovic and colleagues (Gasparovic et al., 2006; Gussew, Erdtel, Hiepe, Rzanny, & Reichenbach, 2012). Exclusion criteria for metabolite failure in fitting the QUEST algorithm included signal-to-noise ratio < 3, full width at half maximum (FWHM) > 0.1 ppm (Wilson et al., 2019), and Cramer–Rao lower bounds (CRLB) > 20%.

### Statistical analysis

Demographic, clinical, and cognitive group differences were examined with chi-square and t-tests, where appropriate. dACC Glu levels for each scan time and group were tested for normality using Kolmogorov–Smirnov tests and outlier detection was performed using the Grubbs’ test. Baseline dACC Glu levels were first compared between HC and FEP using an ANCOVA while controlling for age, sex, and smoking (packs per day). To determine if Glu levels changed over time in the FEP group, we utilized a general linear mixed model with a first-order autoregressive covariance structure, with subjects as a random effect and scan time as a fixed effect while controlling for age, sex, and smoking. Applying a mixed-effects model allows for the use of all observations without list-wise deletion. Exploratory analyses on the identified remitters and nonremitters were also conducted using the same methodology as described above.

We used partial correlations to determine if baseline and change in Glu levels were predictive of treatment response. To determine change in Glu levels over time, we calculated the difference between Week 16 and baseline Glu 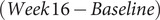 . Baseline and changes in Glu levels and treatment response were then correlated using partial correlations while controlling for age, sex, and packs per day. We used false discovery rate (FDR) to adjust for alpha inflation when appropriate.

Additionally, the above analyses were also repeated with similar covariates and effects, as a completer analysis (only participants with all Glu data points were included).

## Results

### Demographics and clinical data

Demographic data are summarized in Table 1. No significant group differences were observed for sex, age, or parental SES. FEP patients smoked more packs per day compared to HCs. As expected, HCs scored higher on the RBANS than FEP. In patients, the BPRS positive-4 subscale (*t*_82_ = 16.09, *P* < 0.001) and BPRS total scores (*t*_81_ = 14.66, *P* < 0.001) significantly decreased after 16 weeks of treatment, but not for BPRS negative scale scores (*t*_82_ = 0.51, *P* = 0.31).

**Table 1.** Demographics and clinical measures for all participants

	HC (*n* = 117)	FEP (*n* = 113)	*P*-value
Demographic variables			
Age (in years)	23.88 ± 5.21	23.35 ± 5.54	0.46
Sex (M/F)	66/51	71/42	0.32
^a^Parental occupation (SES)	4.5 ± 4.04	5.41 ± 4.63	0.13
No. of smokers (%)	3%	39%	
Smoking (Packs per day)	0.01	0.24	< 0.001
^b^No. of cannabis users (%)	1%	45%	< 0.001
Clinical variables			
^c^Diagnosis			
Schizophrenia	–	53	–
Schizoaffective disorder	–	21	–
Psychosis NOS	–	18	–
Schizophreniform disorder	–	8	–
Brief psychotic disorder	–	5	–
Bipolar disorder w/psychosis	–	4	–
Major depressive disorder w/psychosis	–	3	–
Duration of untreated psychosis (in months)	–	23.25 ± 39.30	–
Pills missed (Week 6)	–	0.74 ± 2.02	–
Pills missed (Week 16)	–	0.99 ± 4.19	–
Dosage in mg. (Week 6)	–	4.27 ± 2.12	–
Dosage in mg. (Week 16)		4.56 ± 2.29	
BPRS (baseline)			
Positive–4	–	14.79 ± 3.89	–
Negative	–	5.46 ± 3.09	–
Total	–	47.84 ± 11.31	–
^d^BPRS (Week 6)			
Positive–4	–	7.59 ± 3.84	–
Negative	–	5.43 ± 2.58	–
Total	–	32.17 ± 8.51	–
^e^BPRS (Week 16)			
Positive–4	–	6.29 ± 2.84	–
Negative	–	5.23 ± 2.61	–
Total	–	29.22 ± 6.00	–
^f^Treatment response (%)	–	74.90 ± 33.23	–
^g^RBANS			
Immediate memory	103.12 ± 14.84	83.26 ± 17.93	< 0.001
Visuospatial/constructional	83.88 ± 13.76	74.76 ± 16.88	< 0.001
Language	97.60 ± 13.94	85.34 ± 16.08	< 0.001
Attention	102.45 ± 14.87	80.81 ± 17.80	< 0.001
Delayed memory	92.01 ± 9.90	77.36 ± 15.88	< 0.001
Total index	93.89 ± 10.74	75.05 ± 15.55	< 0.001
**Scan quality data**			
GM fraction			
Baseline	76.24 ± 4.27	74.16 ± 7.85	0.01
Week 6	75.70 ± 4.35	73.88 ± 4.46	0.01
Week 16	75.47 ± 6.38	73.56 ± 4.58	0.04
WM fraction			
Baseline	9.86 ± 4.45	11.14 ± 7.29	0.11
Week 6	10.50 ± 4.18	10.52 ± 4.23	0.98
Week 16	10.20 ± 5.89	9.93 ± 4.07	0.75
CSF fraction			
Baseline	13.90 ± 3.84	14.70 ± 4.80	0.17
Week 6	13.80 ± 3.66	15.60 ± 4.30	0.004
Week 16	14.34 ± 3.53	16.51 ± 4.21	< 0.001
SNR			
Baseline	14.15 ± 1.55	14.26 ± 1.94	0.63
Week 6	13.98 ± 1.53	14.29 ± 1.44	0.08
Week 16	13.96 ± 1.59	14.25 ± 1.82	0.14
FWHM (in Hz)			
Baseline	2.09 ± 0.56	2.35 ± 0.95	0.07
Week 6	2.07 ± 0.52	2.16 ± 0.64	0.15
Week 16	2.21 ± 1.20	2.34 ± 1.30	0.59

*Note*: Mean ± standard deviation; data available for 229 subjects. Ranks determined from Diagnostic Interview for Genetic Studies where a higher rank (lower numerical value) corresponds to higher socioeconomic status (SES); 222 subjects; 112 subjects; 93 subjects; 84 subjects; 84 subjects; 207 subjects; RBANS, Repeatable Battery for the Assessment of Neuropsychological Status; BPRS, Brief Psychiatric Rating Scale; *P*-values are from χ2 and independent samples *t-*tests for differences between groups.

Out of the 84 patients with BPRS positive-4 subscale scores at 16 weeks, 61 patients (73%) met remission criteria, and 23 (27%) patients were in nonremission status. Remission and nonremission patients did not differ on age, sex, smoking, positive symptoms at baseline, and cognition; but differed on parental SES, duration of untreated psychosis, and negative symptoms at baseline (Table 2).

**Table 2.** Demographics and clinical measures of remission and nonremission groups

	Remission (*n* = 61)	Nonremission (*n* = 23)	*P*-value
Demographic variables			
Age (in years)	23.62 ± 5.76	25.17 ± 6.12	0.28
Sex (M/F)	36/25	16/7	0.38
Parental occupation (SES)	4.75 ± 4.55	7.04 ± 5.63	0.06
No. of smokers (%)	3%	39%	
Smoking (packs per day)	0.23	0.26	0.79
No. of cannabis users (%)	42%	33%	0.48
Clinical variables			
Diagnosis			
Schizophrenia	34	12	–
Schizoaffective disorder	13	5	–
Psychosis NOS	7	2	–
Schizophreniform disorder	2	1	–
Brief psychotic disorder	1	1	–
Bipolar disorder w/psychosis	3	1	–
Major depressive disorder w/psychosis	1	1	–
Duration of untreated psychosis (in months)	19.25 ± 36.05	37.23 ± 50.35	0.05
Pills missed (Week 6)	0.68 ± 1.88	0.93 ± 2.58	0.70
Pills missed (Week 16)	1.08 ± 4.90	0.86 ± 2.07	0.87
Dosage in mg. (Week 6)	4.21 ± 2.09	4.95 ± 2.09	0.17
Dosage in mg. (Week 16)	4.28 ± 2.16	5.37 ± 2.53	0.08
BPRS (baseline)			
Positive–4	14.82 ± 3.72	15.17 ± 4.60	0.72
Negative	4.97 ± 2.96	6.70 ± 3.75	0.03
Total	47.74 ± 11.21	49.91 ± 11.87	0.44
BPRS (Week 6)			
Positive–4	6.59 ± 2.46	9.57 ± 5.72	0.001
Negative	5.09 ± 2.26	6.52 ± 3.23	0.03
Total	30.00 ± 5.54	37.65 ± 11.57	< 0.001
BPRS (Week 16)			
Positive–4	5.36 ± 1.87	8.74 ± 3.48	< 0.001
Negative	4.85 ± 2.07	6.22 ± 3.55	0.03
Total	26.87 ± 4.71	35.35 ± 4.49	< 0.001
Treatment response (%)	84.37 ± 24.28	49.78 ± 40.61	< 0.001
RBANS			
Immediate memory	82.72 ± 16.70	79.90 ± 18.40	0.52
Visuospatial/constructional	75.77 ± 17.03	75.62 ± 18.11	0.97
Language	85.67 ± 16.33	81.19 ± 16.42	0.28
Attention	79.92 ± 15.81	81.67 ± 22.52	0.70
Delayed memory	78.27 ± 13.92	75.10 ± 17.63	0.41
Total index	75.63 ± 15.10	73.95 ± 17.85	0.68
**Scan quality data**			
GM fraction			
Baseline	74.81 ± 5.49	72.61 ± 4.52	0.09
Week 6	74.52 ± 4.64	72.97 ± 4.38	0.21
Week 16	73.86 ± 4.75	72.76 ± 4.21	0.39
WM fraction			
Baseline	10.36 ± 4.46	11.22 ± 4.04	0.42
Week 6	10.18 ± 4.46	10.46 ± 3.72	0.81
Week 16	9.66 ± 3.89	10.51 ± 4.65	0.45
CSF fraction			
Baseline	14.84 ± 4.54	16.16 ± 5.93	0.28
Week 6	15.30 ± 3.95	16.57 ± 4.95	0.26
Week 16	16.48 ± 4.18	16.73 ± 4.49	0.83
SNR			
Baseline	14.06 ± 2.14	14.34 ± 1.55	0.56
Week 6	14.03 ± 1.34	14.81 ± 2.13	0.06
Week 16	14.35 ± 1.84	14.40 ± 1.76	0.92
FWHM (in Hz)			
Baseline	2.34 ± 0.95	2.20 ± 0.52	0.51
Week 6	2.26 ± 0.71	2.28 ± 1.07	0.94
Week 16	2.41 ± 1.46	2.34 ± 1.26	0.85

*Note*: Mean ± standard deviation. Ranks determined from Diagnostic Interview for Genetic Studies where a higher rank (lower numerical value) corresponds to higher socioeconomic status (SES). RBANS, Repeatable Battery for the Assessment of Neuropsychological Status; BPRS, Brief Psychiatric Rating Scale; *P*-values are from χ2 and independent samples *t-*tests for differences between groups.

### Between-group baseline and longitudinal glutamate levels

**Figure 1.**
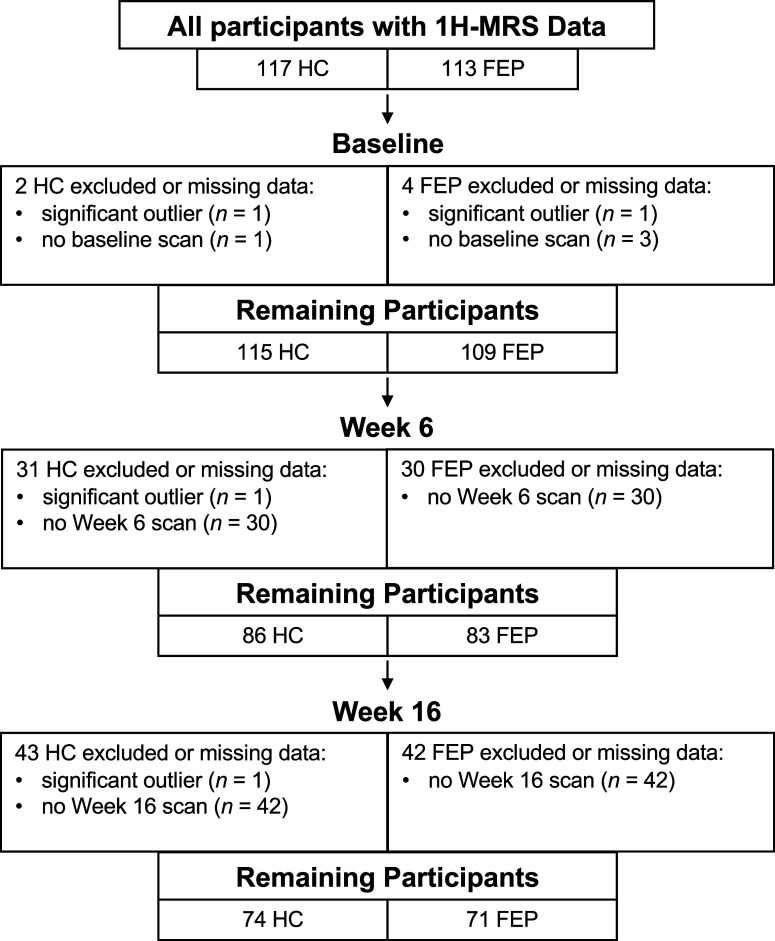
CONSORT flow chart for healthy controls (HC) and first-episode psychosis patients (FEP).

Our total number of participants used for this study with available data (i.e., who had at least one scan time) was 232 (HC = 117; FEP = 113; Figure 1). All dACC Glu levels for each scan time met assumptions of normality (all *p*’s n.s.). After accounting for participants who had either missing data or were statistically significant outliers (based on Grubbs’ test), our numbers for participants varied between groups (Figure 1): 224 for baseline (HC = 115; FEP = 109), 169 for Week 6 (HC = 86; FEP = 83), and 145 (HC = 74; FEP = 71) for Week 16. Given that FWHM (*F*_2, 242.54_ = 0.47, *P* = 0.63) and SNR (*F*_2, 263.43_ = 0.47, *P* = 0.62) did not show any significant group × scan time interactions, these were not included as additional covariates.

**Figure 2.**
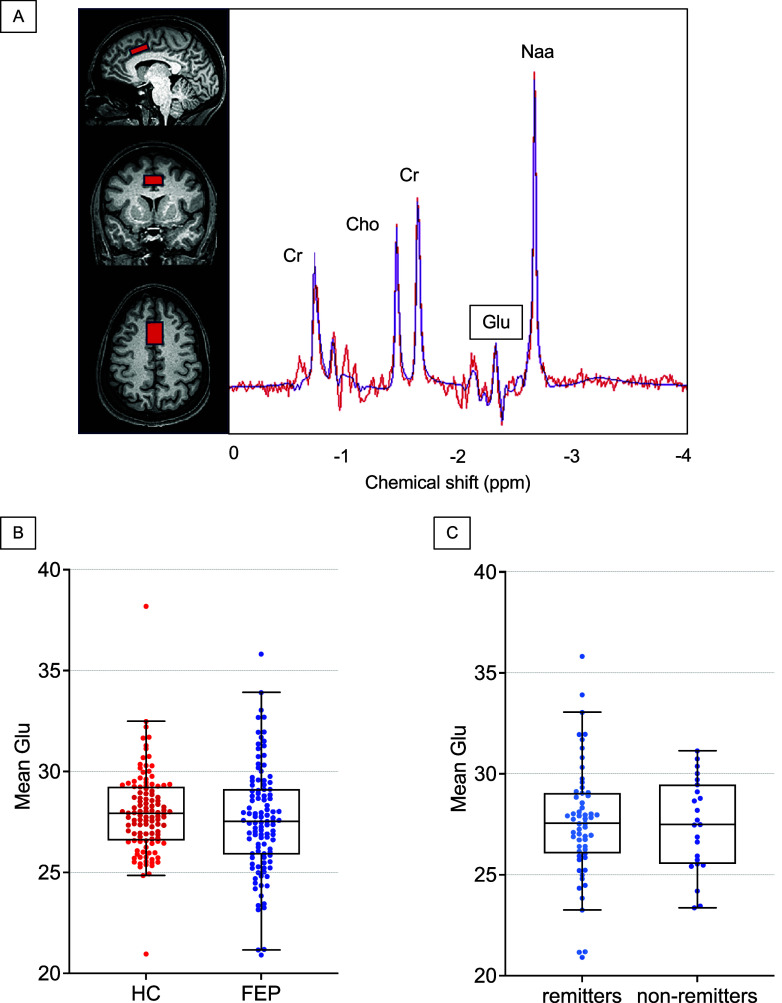
(A) Voxel placement in the dorsal anterior cingulate cortex and example spectrum. The red line represents averaged spectra, and the black line is an overlay of the spectral fit; (B) boxplot depicting baseline Glu concentration for each group. Each individual dot is a data point from a single subject and black lines indicate medial value for each group; (C) boxplot depicting baseline Glu concentration for remitters and non-remitters. Each individual dot is a data point from a single subject and black lines indicate medial value for each group. Glu, glutamate; ppm, parts per million; HC, healthy controls; FEP, first-episode psychosis.

**Figure 3.**
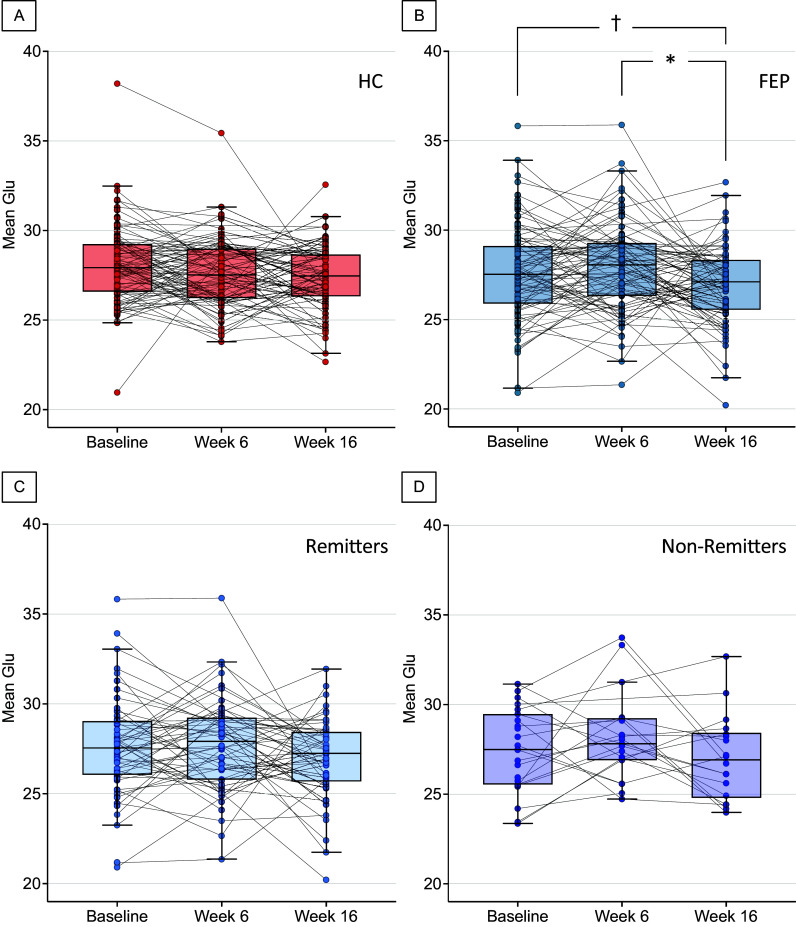
Boxplots depicting Glu levels for (A) HC (in red); (B) FEP group (in blue); (C) remitters (in light blue); and (D) non-remitters (in light purple) at each scan time. Each individual dot is a data point from a single subject and black lines indicate medial value. Glu, glutamate; HC, healthy controls; FEP, first-episode psychosis. ^†^*P*_FDR_ < 0.10, *P_FDR_ < 0.05.

dACC Glu levels at baseline did not differ between HC and FEP groups after controlling for age, sex, and smoking (*F*_1, 219_ = 2.12, Cohen’s *d* = 0.16, *P* = 0.15, Figure 2B, Table 3). Similarly, dACC Glu levels at baseline did not differ between remitters and nonremitters (*F*_1, 77_ = 0.15, Cohen’s *d* = 0.05, *P* = 0.70, Figure 2C). Results from linear mixed model showed a significant effect of scan time within FEP patients (*F*_2, 149.03_ = 4.17, *P* = 0.02, Figure 3A) and follow-up tests adjusting for alpha inflation revealed no significant difference between Baseline and Week 6 (*CI*_95%_ = −0.94, 0.29, Cohen’s *d* = 0.11, *P*_FDR_ = 0.30), a marginal one between Baseline and Week 16 (*CI*_95%_ = −0.04, 1.38, Cohen’s *d* = 0.20, *P*_FDR_ = 0.09), and a significant difference between Week 6 and Week 16 (*CI*_95%_ = 0.31, 1.68, Cohen’s *d* = 0.30, *P*_FDR_ = 0.01). A validation analysis using the HC group was implemented and revealed a marginal effect of scan time (*F*_2, 164.20_ = 3.0, *P* = 0.052, Figure 3B). Follow-up tests comparing each scan time revealed no difference between Baseline and Week 6 (*CI*_95%_ = −0.11, 0.83, Cohen’s *d* = 0.18, *P*_FDR_ = 0.19), a marginal difference between Baseline and Week 16 (*CI*_95%_ = 0.12, 1.19, Cohen’s *d* = 0.25, *P*_FDR_ = 0.054), or no difference between Week 6 and Week 16 (*CI*_95%_ = −0.23, 0.82, Cohen’s *d* = 0.10, *P*_FDR_ = 0.28). Finally, no significant effects of scan time were found when examining longitudinal Glu levels in remitters (*F*_2, 106.13_ = 1.8, *P* = 0.17, Figure 3C) and nonremitters (*F*_2, 35.27_ = 1.9, *P* = 0.16, Figure 3D; Table 4).

**Table 3.** Data on dACC Glu and CRLB in HC and FEP patients

	HC		FEP	
Scan time	Mean Glu ± SD	Mean CRLB ± SD	Mean Glu ± SD	Mean CRLB ± SD
Baseline	27.97 ± 2.04	4.63 ± 0.87	27.60 ± 2.66	5.01 ± 1.20
Week 6	27.57 ± 1.93	4.60 ± 0.50	27.91 ± 2.51	4.89 ± 0.86
Week 16	27.34 ± 1.85	4.59 ± 0.48	26.95 ± 2.27	5.40 ± 1.96

Abbreviations: dACC, dorsal anterior cingulate cortex; HC, healthy control; FEP, first-episode psychosis; Glu, glutamate; CRLB, Cramer–Rao lower bounds; SD, standard deviation.

**Table 4.** Data on dACC Glu and CRLB in remitters and nonremitters

	Remitters		Nonremitters	
Scan time	Mean Glu ± SD	Mean CRLB ± SD	Mean Glu ± SD	Mean CRLB ± SD
Baseline	27.58 ± 2.86	5.12 ± 1.47	27.46 ± 2.30	4.95 ± 0.85
Week 6	27.76 ± 2.58	4.90 ± 0.79	28.17 ± 2.48	5.09 ± 1.16
Week 16	26.96 ± 2.25	5.40 ± 0.48	27.03 ± 2.37	5.43 ± 1.68

Abbreviations: dACC, dorsal anterior cingulate cortex; Glu, glutamate; CRLB, Cramer–Rao lower bounds; SD, standard deviation.

### Glutamate levels and symptom severity

**Figure 4.**
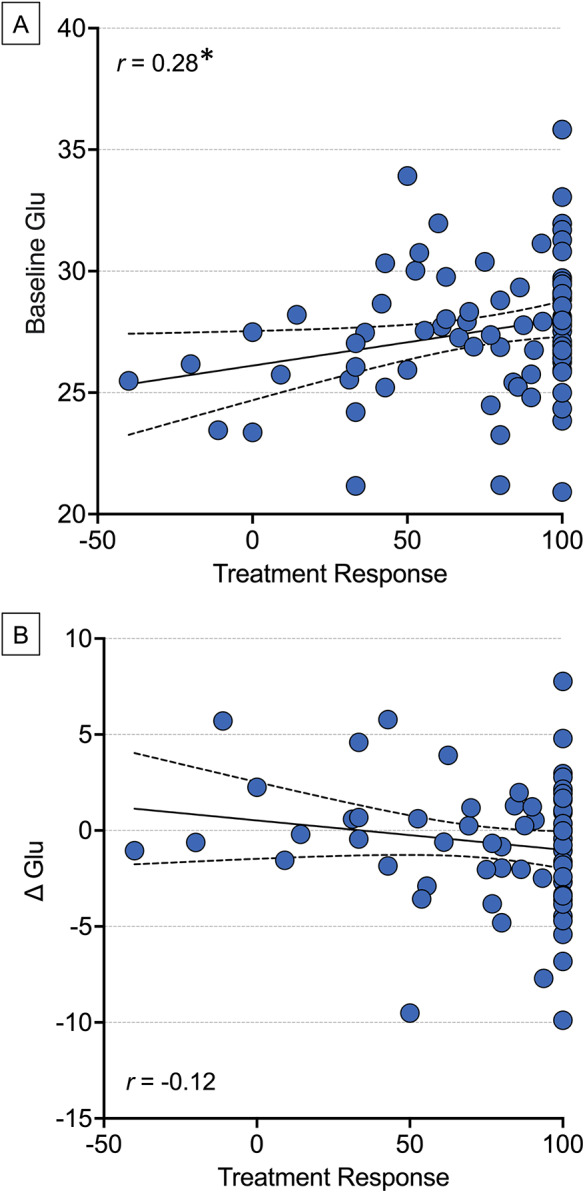
Scatterplots of (A) baseline Glu levels and (B) Δ Glu versus treatment response for FEP patients. Dotted lines indicate 95% confidence bands. GLU, glutamate. *P_FDR_ < 0.05.

Partial correlation analyses showed a significant correlation between baseline Glu levels and treatment response (*r*_77_ = 0.28, *P*_FDR_ = 0.03, Figure 4A), but change in Glu levels was not correlated with treatment response (*r*_63_ = −0.20, *P*_FDR_ = 0.12, Figure 4B).

### Completer analysis

Our total number of participants used with all data points was 126 (HC = 63; FEP = 63). No significant group differences were observed for sex (HC = 37 M/26F; FEP = 39 M/24F), age (HC = 24.17 years; FEP = 23.48 years), or parental SES. Significant differences remained in FEP patients in packs per day and all RBANS subscales. In patients, the BPRS positive-4 subscale (*t*_61_ = 14.05, *P* < 0.001) and BPRS total scores (*t*_61_ = 12.71, *P* < 0.001) significantly decreased after 16 weeks of treatment but not for BPRS negative scale scores (*t*_62_ = 0.20, *P* = 0.39).

dACC Glu levels at baseline did not differ between HC and FEP groups after controlling for age, sex, and smoking (*F*_1, 121_ = 5.94, Cohen’s *d* = 0.18, *P* = 0.25, Supplementary Figure 1A). Similarly, dACC Glu levels at baseline did not differ between remitters and nonremitters (*F*_1, 57_ = 0.25, Cohen’s *d* = 0.05, *P* = 0.62, Supplementary Figure 1B). Results from linear mixed model showed a significant effect of scan time within FEP patients (*F*_2, 94.54_ = 4.41, *P* = 0.02, Supplementary Figure 2A) and follow-up tests adjusting for alpha inflation revealed no significant difference between Baseline and Week 6 (*CI*_95%_ = −1.13, 0.37, Cohen’s *d* = 0.12, *P*_FDR_ = 0.32), no significant difference between Baseline and Week 16 (*CI*_95%_ = −0.08, 1.56, Cohen’s *d* = 0.23, *P*_FDR_ = 0.11), and a significant difference between Week 6 and Week 16 (*CI*_95%_ = 0.37, 1.87, Cohen’s *d* = 0.35, *P*_FDR_ = 0.01). A validation analysis using the HC group was implemented and revealed a marginal effect of scan time (*F*_2, 93.01_ = 2.78, *P* = 0.07, Supplementary Figure 2B). Finally, no significant effect of scan time was found when examining longitudinal Glu levels in remitters (*F*_2, 65.25_ = 1.98, *P* = 0.15, Supplementary Figure 2C) but a marginal difference was found in nonremitters (*F*_2, 27.83_ = 2.79, *P* = 0.08, Supplementary Figure 2D).

Partial correlation analyses showed a significant correlation between baseline Glu levels and treatment response (*r*_58_ = 0.31, *P*_FDR_ = 0.04, Supplementary Figure 3A), but change in Glu levels was not correlated with treatment response (*r*_57_ = −0.20, *P*_FDR_ = 0.12, Supplementary Figure 3B).

## Discussion

To the best of our knowledge, this is the first study to examine over a 100 antipsychotic-naïve FEP patients using 1H-MRS spectroscopy to measure Glu. First, there were no significant group differences found between HC and FEP prior to antipsychotic treatment on Glu levels. Second, positive symptomology significantly decreased after 16 weeks of treatment. Third, examining Glu levels over time exclusively in the FEP group using linear mixed models showed a significant reduction in Glu from Week 6 to Week 16 and a marginal one from Baseline to Week 16. This effect was weaker in HC. Furthermore, Baseline Glu, but no changes in Glu, were positively and significantly associated with treatment response in FEP. Finally, these primary findings remained stable after a completer analysis.

Similar to Li et al. (2020) and Fan et al. (2024), we found no difference in Glu levels in medication-naïve FEP compared to HC. Our findings also concur with large meta-analyses of Glu levels in SZ that found lower MFC Glu in patients, but no difference in Glu levels when the analysis was limited to medication-naïve/unmedicated patients (Merritt et al., 2023; Smucny, Carter, & Maddock, 2021). Greater Glu variability in FEP compared to HC (as confirmed by Levene’s test; *P* = 0.009) was also found, and this is rather noteworthy as this same effect was reported by Merrit and colleagues. Furthermore, our effect size between baseline medication-naïve FEP compared to HC (0.16) is very similar to that of Smucny and colleagues for unmedicated patients (0.15), which adds merit to these consistencies. This contrasts with Bojesen who, in a cohort of medication-naïve FEP like ours, found lower Glu levels compared to HC before treatment (Bojesen et al., 2020). Several factors could have influenced these discrepant results. Increased variability in Glu levels in patients and proportion of males can affect Glu measurement (Merritt et al., 2023) as well as methodological variables such as field strength, echo time (Smucny, Carter, & Maddock, 2021), and voxel placement and size within the ACC (Fan et al., 2024; Nakahara et al., 2022). In addition, shifts in bioenergetic regulation that are affected by genetic and environmental factors could impact downstream levels of Glu (Stein, Zhu, Du, & Ongur, 2023).

We found that Glu levels decrease even after a short exposure to APD, more specifically in this study, Glu levels decrease between week 6 and week 16. This agrees with Egerton et al. (2018) study where Glu was lower at 4 weeks than at baseline, and with a large meta-analysis where ACC Glu was reduced in medicated but not in medication-naïve patients (Merritt et al., 2023). Five studies did not show a significant change in ACC Glu levels over time (Bojesen et al., 2020; Bustillo et al., 2010; Fan et al., 2024; Kraguljac et al., 2019; Zahid et al., 2022), but there was much variability in patients’ medication status and the length of APD treatment. A possible explanation for these changes in Glu over time is that risperidone may have suppressed NMDA blockade-mediated glutamate release through 5-HT receptors and increased AMPA receptors (which are glutamate-gated ion channels) in prefrontal cortex and its projections, as it has been previously shown in rats (Choi, Gardner, & Tarazi, 2009; Roenker et al., 2011). These findings add support to the NMDA and AMPA receptor changes in the brain may contribute to the psychopharmacological actions of risperidone in psychosis.

In addition, treatment response (as measured as % change in positive symptomology from baseline after 16 weeks of treatment) was associated with dACC Glu baseline levels. This association suggests that those patients with initial higher dACC Glu levels showed the largest decrease in positive symptoms. Our results concur with those of Li et al. (2020) who show that higher baseline ACC Glu was associated with better treatment response but in contrast with Egerton et al. (2018) showing the opposite results. Egerton’s results were based on subjects’ recruitment at several sites, smaller n and a measure of glutamate scaled to creatine. Our attempt at characterizing remission status in our study did not yield a significant effect on dACC Glu at baseline or throughout the duration of the trial, which is consistent with Egerton et al. (2023) and Fan et al. (2024). While some studies have reported significant remission effects on Glu levels, these have varied in terms of when remission was established (4 versus 26 weeks), prior exposure to antipsychotics, and diagnosis. Though a more systematical approach to characterize response to antipsychotics has yet to be developed, examining response to treatment in the form of a spectrum may reveal a more accurate clinical picture of the illness as a progressive disorder. In addition to the patient’s response to treatment variability, other variables such as age, diagnosis, symptom severity, and level of functioning may account for heterogeneity within the illness (Merritt et al., 2021). Overall, our findings suggest that APDs can influence Glu within a period of 16 weeks, which has been deemed as an optimal window for symptom alleviation using APDs (Gallego et al., 2011; Phahladira et al., 2020).

Brain functional connectivity measured with functional MRI (fMRI) during a resting state has been shown by several to also predict treatment response to antipsychotic medications (Mehta et al., 2021; Nelson, Kraguljac, Maximo, Armstrong, & Lahti, 2022, 2023; Sarpal et al., 2016). Identifying those who are not likely to respond to traditional treatment prior to treatment within the first 16 weeks and who should be targeted for treatment with clozapine might come from a combination of imaging measurements, such as baseline Glu levels and functional connectivity patterns.

Strengths and limitations of this study are the following: we only enrolled medication-naïve FEP patients with very limited exposure to antipsychotics (no more than 5 days of lifetime antipsychotic exposure), allowing us to mitigate any medication or illness chronicity effects. Another strength is that our study had three time points quite close to one another which allowed us to determine any inflection point throughout the treatment. We acknowledge that the lack of a placebo-control group did not allow us to determine whether the observed Glu changes are a direct result of medication treatment. We made this choice because withholding treatment is ethically not permissible. Similarly, although antipsychotic dosage and pill count were recorded at each timepoint, compliance was not confirmed through antipsychotic plasma levels. Another limitation was the attrition rate in our participants which was exacerbated by the COVID-19 pandemic. Nonetheless, our primary findings showed robustness across completer analysis, which gives assurance that our primary results were not driven by attrition bias. Finally, exposure to cannabis may affect brain functioning and it is considered as one of the major risk factors for developing psychosis and consequently highly clinically relevant. Excluding patients using cannabis would have inadvertently biased our sample and limited the generalizability of our results.

We report a progressive decrease of Glu levels over a period of 16 weeks after initiation of treatment in a large sample of medication-naïve FEP patients and a baseline Glu level association with a reduction in positive symptomology, suggestive of a potential mechanism of APD action. Measurements of Glu levels might contribute to identifying those who are not likely to respond to traditional antipsychotics and should be targeted for clozapine treatment.

## Supporting information

Maximo et al. supplementary material Maximo et al. supplementary material

## Supplementary material

The supplementary material for this article can be found at http://doi.org/10.1017/S0033291724003386.

## Clinical trial registration

Trajectories of Treatment Response as Window into the Heterogeneity of Psychosis: A Longitudinal Multimodal Imaging Study, NCT03442101 (https://clinicaltrials.gov/ct2/show/NCT03442101). Glutamate, Brain Connectivity and Duration of Untreated Psychosis (DUP), NCT02034253 (https://clinicaltrials.gov/ct2/show/NCT02034253).
