# Supplementary material for: Changes in glutamate levels in anterior cingulate cortex following 16 weeks of antipsychotic treatment in antipsychotic-naïve first-episode psychosis patients
Source: Psychol Med. 2025 Feb 10;55:e35. doi: 10.1017/S0033291724003386 (PMC12017365; doi:10.1017/S0033291724003386)
Supplement: Maximo et al. supplementary material [file S0033291724003386sup001.pptx]

## Slide 1
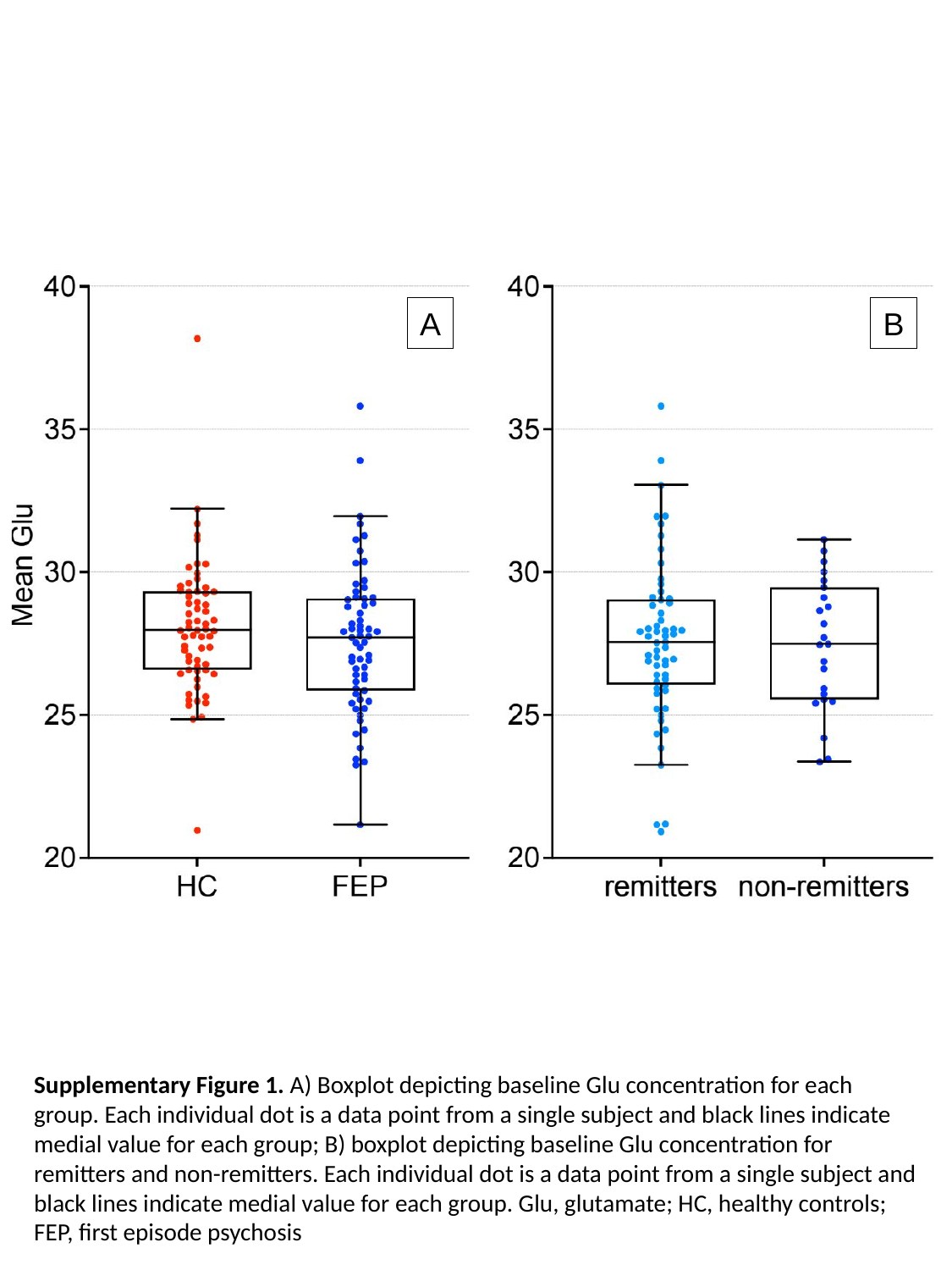

A
B
Supplementary Figure 1. A) Boxplot depicting baseline Glu concentration for each group. Each individual dot is a data point from a single subject and black lines indicate medial value for each group; B) boxplot depicting baseline Glu concentration for remitters and non-remitters. Each individual dot is a data point from a single subject and black lines indicate medial value for each group. Glu, glutamate; HC, healthy controls; FEP, first episode psychosis

## Slide 2
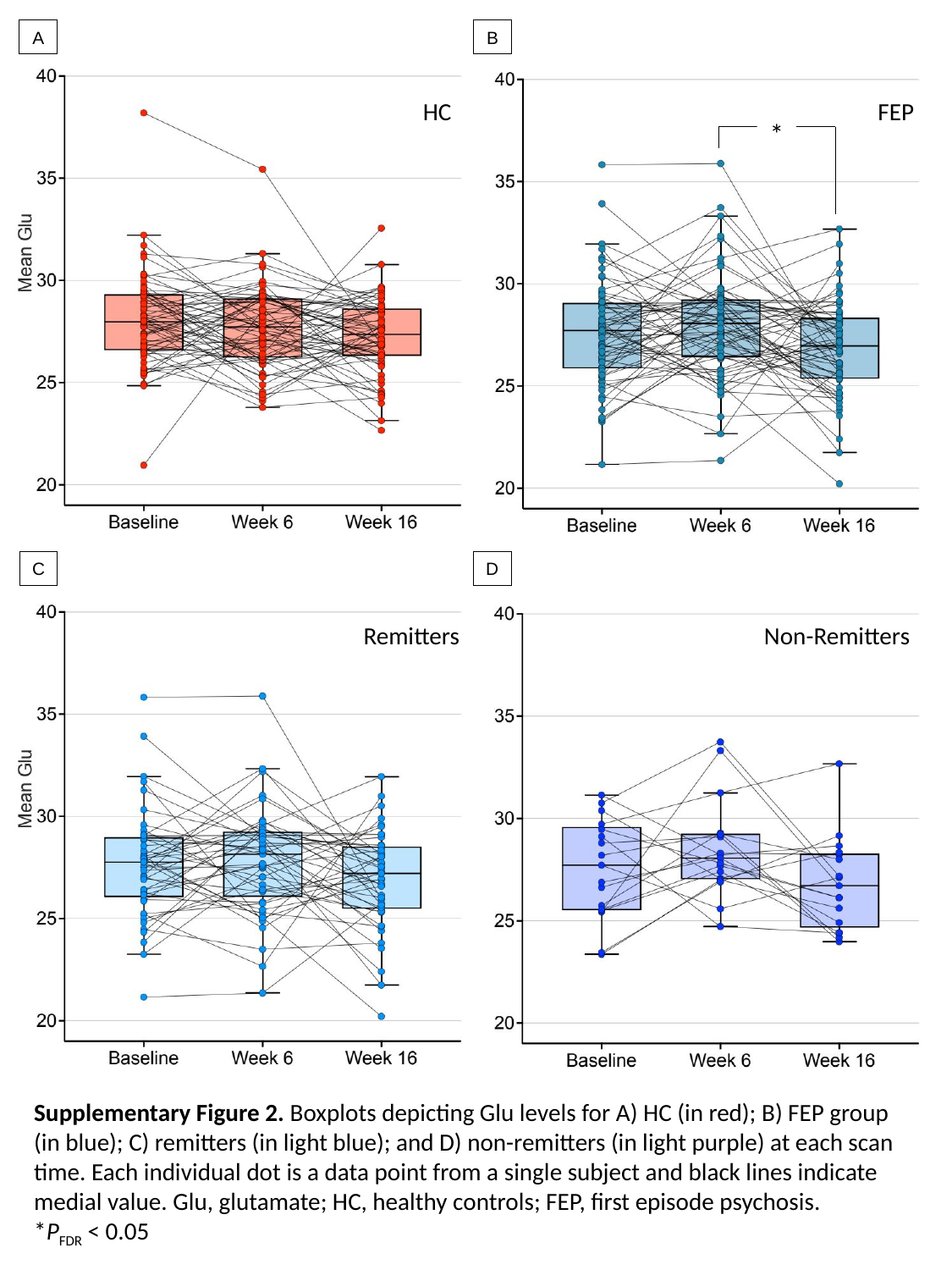

B
A
FEP
HC
*
C
D
Remitters
Non-Remitters
Supplementary Figure 2. Boxplots depicting Glu levels for A) HC (in red); B) FEP group (in blue); C) remitters (in light blue); and D) non-remitters (in light purple) at each scan time. Each individual dot is a data point from a single subject and black lines indicate medial value. Glu, glutamate; HC, healthy controls; FEP, first episode psychosis.
*PFDR < 0.05

## Slide 3
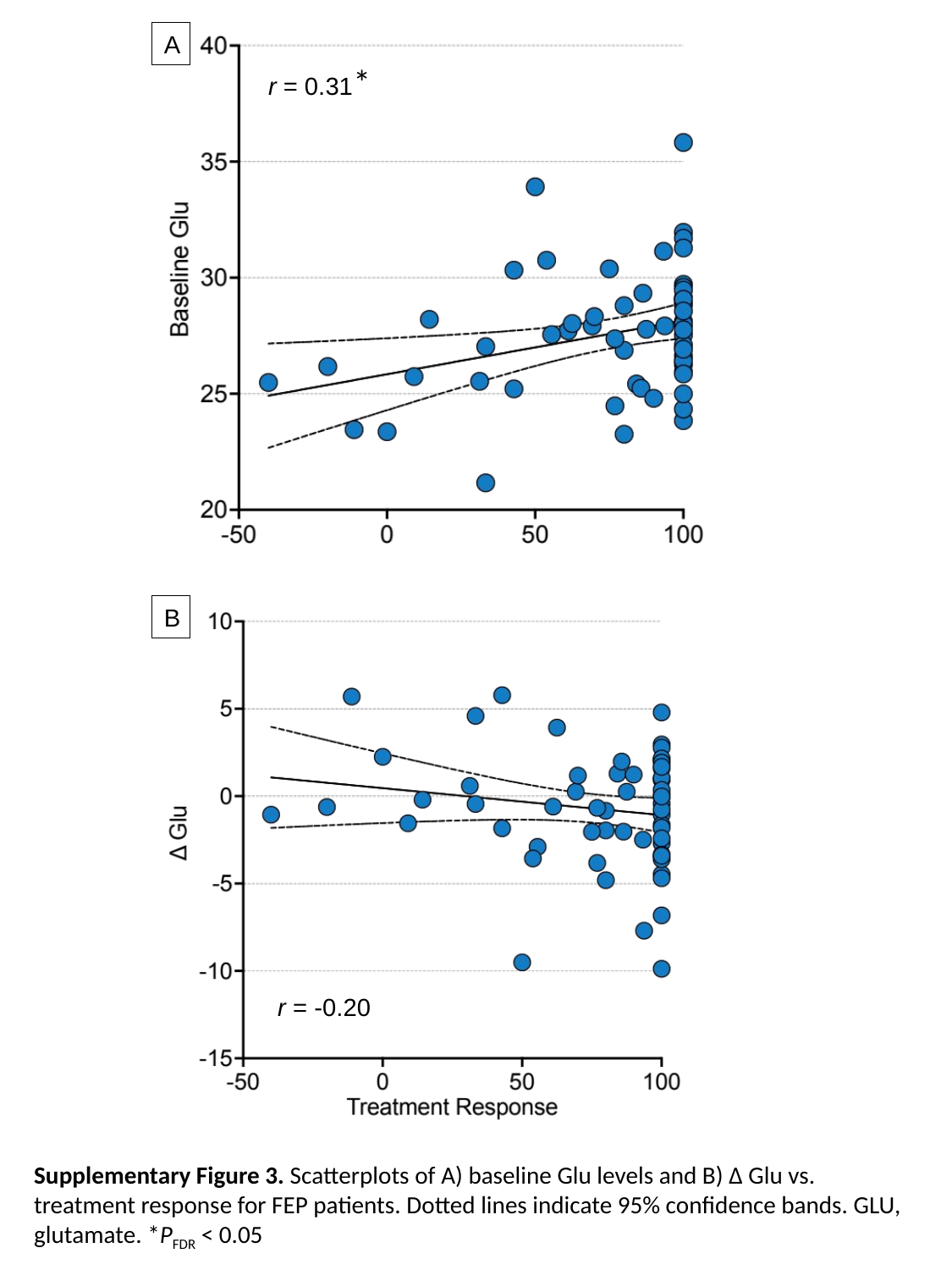

A
r = 0.31*
B
r = -0.20
Supplementary Figure 3. Scatterplots of A) baseline Glu levels and B) Δ Glu vs. treatment response for FEP patients. Dotted lines indicate 95% confidence bands. GLU, glutamate. *PFDR < 0.05
